# PIM protein kinases regulate the level of the long noncoding RNA H19 to control stem cell gene transcription and modulate tumor growth

**DOI:** 10.1002/1878-0261.12662

**Published:** 2020-04-01

**Authors:** Neha Singh, Sathish K. R. Padi, Jeremiah J. Bearss, Ritu Pandey, Koichi Okumura, Himisha Beltran, Jin H. Song, Andrew S. Kraft, Virginie Olive

**Affiliations:** ^1^ University of Arizona Cancer Center University of Arizona Tucson AZ USA; ^2^ Department of Cellular and Molecular Medicine University of Arizona Tucson AZ USA; ^3^ Department of Physiology University of Arizona Tucson AZ USA; ^4^ Department of Medical Oncology Dana‐Farber Cancer Institute Boston MA USA; ^5^ Department of Medicine University of Arizona Tucson AZ USA

**Keywords:** androgen deprivation therapy resistance, H19, Pim kinase, prostate cancer, SOX2, T‐ALL

## Abstract

The proviral integration site for Moloney murine leukemia virus (PIM) serine/threonine kinases have an oncogenic and prosurvival role in hematological and solid cancers. However, the mechanism by which these kinases drive tumor growth has not been completely elucidated. To determine the genes controlled by these protein kinases, we carried out a microarray analysis in T‐cell acute lymphoblastic leukemia (T‐ALL) comparing early progenitor (ETP‐ALL) cell lines whose growth is driven by PIM kinases to more mature T‐ALL cells that have low PIM levels. This analysis demonstrated that the long noncoding RNA (lncRNA) H19 was associated with increased PIM levels in ETP‐ALL. Overexpression or knockdown of PIM in these T‐ALL cell lines controlled the level of H19 and regulated the methylation of the H19 promoter, suggesting a mechanism by which PIM controls H19 transcription. In these T‐ALL cells, the expression of PIM1 induced stem cell gene expression (SOX2, OCT‐4, and NANOG) through H19. Identical results were found in prostate cancer (PCa) cell lines where PIM kinases drive cancer growth, and both H19 and stem cell gene levels. Small molecule pan‐PIM inhibitors (PIM‐i) currently in clinical trials reduced H19 expression in both of these tumor types. Importantly, the knockdown of H19 blocked the ability of PIM to induce stem cell genes in T‐ALL cells, suggesting a novel signal transduction cascade. In PCa, increases in SOX2 levels have been shown to cause both resistance to the androgen deprivation therapy (ADT) and the induction of neuroendocrine PCa, a highly metastatic form of this disease. Treatment of PCa cells with a small molecule pan‐PIM‐i reduced stem cell gene transcription and enhanced ADT, while overexpression of H19 suppressed the ability of pan‐PIM‐i to regulate hormone blockade. Together, these results demonstrate that the PIM kinases control the level of lncRNA H19, which in turn modifies stem cell gene transcription regulating tumor growth.

## Graphical abstract

The present study demonstrates that in T‐ALL and prostate cancer, proviral integration site for Moloney murine leukemia virus (PIM) kinase induction increases long noncoding RNA‐H19 that in turn regulates stem cell genes. In resistant T‐ALL, PIM1/H19 signaling restores partial sensitivity to pan‐PIM inhibitors (PIM‐i). In prostate cancer, elevated PIM1 contributes to androgen deprivation therapy resistance reversed by a combination with PIM‐i. These data suggest a novel and clinically relevant pathway controlled by PIM kinases.

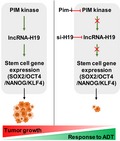

**Abbreviations**
ADT: androgen deprivation therapy
ALL: acute lymphoblastic leukemia
AR: androgen receptor
BPH: benign prostate hyperplasia
DMR: differentially methylated region
Dox: doxycycline
Enza: enzalutamide
ES cells: embryonic stem cells
ETP‐ALL: early progenitor‐type acute lymphoblastic leukemia
HP1: heterochromatin protein 1γ
ICR: imprinting control region
IRS1: insulin receptor substrate 1
KD: knockdown
KiD: kinase‐dead
KLF4: Krüppel‐like factor 4
lncRNA: long noncoding RNA
NEPC: neuroendocrine prostate cancer
OCT‐4: octamer‐binding transcription factor 4
PCa: prostate cancer
PIM: proviral integration site for Moloney murine leukemia virus
PIM‐i: PIM inhibitor
PRC2: polycomb repressor complex 2
qPCR: quantitative PCR
SEM: standard error of the mean
SOX2: SRY (sex‐determining region Y)‐box 2
T‐ALL: T‐cell acute lymphoblastic leukemia
WB: western blot
WT: wild‐type

## 1 Introduction

The proviral integration site for Moloney murine leukemia virus (PIM) protein kinase family, comprising three serine/threonine protein kinases, PIM1, PIM2, and PIM3, has been implicated in cancer initiation and progression (Brault *et al.*, 2010; Cuypers *et al.*, 1984). PIM kinases are elevated in hematopoietic neoplasms including myeloid leukemia, myeloma, and lymphoma (Alizadeh *et al.*, 2000; Cohen *et al.*, 2004; Wingett *et al.*, 1996), and play an important role in prostate tumorigenesis (Chen *et al.*, 2005; Shah *et al.*, 2008). These enzymes have been associated with aggressive prostate tumor growth (Cibull *et al.*, 2006). PIM kinases control tumor cellular metabolism, protein translation, cell growth, and division, and play a clear role in the resistance to PI3K/AKT‐directed therapies in breast cancer (Braso‐Maristany *et al.*, 2016; Le *et al.*, 2016) and prostate cancer (PCa) (Padi *et al.*, 2019; Song *et al.*, 2018). Although many of the specific targets and pathways that are regulated by the PIM kinases to drive cancer growth and progression have been examined (de Bock *et al.*, 2018; Malone *et al.*, 2019), investigating other potential mechanisms will provide new insights into novel therapeutic targets.

A stem cell gene expression signature has been shown to characterize poorly differentiated tumors from several types of human cancer (Ben‐Porath *et al.*, 2008). Tumor‐initiating cells expressing pluripotency factors, such as NANOG, octamer‐binding transcription factor 4 (OCT‐4), and SRY (sex‐determining region Y)‐box 2 (SOX2), play a significant role in the induction and maintenance of malignancy. Immortalized PCa epithelial cell cultures and DU145 human prostatosphere cells have been shown to express these stem cell genes (Gu *et al.*, 2007; Rybak *et al.*, 2011), which are enriched in high‐Gleason grade PCa (Mathieu *et al.*, 2011), and predict the poorest overall survival (Markert *et al.*, 2011). PIM1 transcriptional levels in embryonic stem (ES) cells are controlled by STAT3 and leukemia‐inhibitory factor, which are known to regulate stem cell genes (Aksoy *et al.*, 2007). When ES cells are fused to fibroblasts, the addition of IL‐6 to stimulate these cultures upregulates PIM1 levels, which then cooperates with OCT‐4, SOX2, and Krüppel‐like factor 4 (KLF4) to increase induced pluripotent stem cell frequency (Brady *et al.*, 2013). Consistent with a role in stem cells, transgenic mice overexpressing PIM1 upregulate hematopoietic stem/progenitor cell proliferation, while PIM1 knockout mice have impaired long‐term hematopoietic repopulating capacity (An *et al.*, 2013). Together, these findings suggest that the PIM kinases could regulate stem cell genes to control cancer growth.

We demonstrate that the PIM1 protein kinase induces the long noncoding RNA (lncRNA) H19 expression and promotes induction of a stem cell signature in both T‐cell acute lymphoblastic leukemia (T‐ALL) and PCa cells. The lncRNA H19 is highly expressed in embryonic tissue and placenta and repressed after birth (Pachnis *et al.*, 1988; Poirier *et al.*, 1991), but is highly re‐expressed in multiple cancers including both hematopoietic (Takeuchi *et al.*, 2007) and solid tumors including breast (Adriaenssens *et al.*, 1998), esophageal (Hibi *et al.*, 1996), bladder (Ariel *et al.*, 1995; Elkin *et al.*, 1995), lung (Kondo *et al.*, 1995), and endometrial and cervical (Lee *et al.*, 2003) cancers. H19 is encoded by the IGF2/H19‐imprinted gene cluster located on human chromosome 11p15 (Zemel *et al.*, 1992), and its transcription is controlled by differentially methylated regions (DMRs) of the upstream DNA called ‘imprinting control regions’ (ICR). When hypomethylated, four specific DMRs upstream of H19 start site bind the transcription factor CCCTC‐binding factor, which acts as insulator preventing IGF2 promoter activation and enhancing H19 transcription (Phillips and Corces, 2009). Here, we show that PIM1 induces epigenetic changes in DNA methylation in the control regions of H19, suggesting a mechanism by which it regulates the transcription of this lncRNA. The inhibition of pan‐PIM kinase activity and the knockdown (KD) of H19 sensitize PCa cells to treatment with antiandrogen enzalutamide (Enza). In PCa and its highly aggressive variant neuroendocrine PCa (NEPC) (Davies *et al.*, 2018), which expresses elevated H19 levels as compared to adenocarcinoma (Ramnarine *et al* *.*, 2018), the combination of pan‐PIM kinase inhibitors with concomitant H19 KD reduces tumor growth. These results delineate a unique signaling cascade driven by the PIM kinases that involves lncRNA and stem cell genes to regulate tumor growth.

## 2 Materials and methods

### 2.1 Cell culture

PC3, LNCaP, and DU145 were purchased from the American Type Culture Collection (ATCC, Baltimore, MD, USA); PC3‐LN4 and human prostate fibroblast cell line BHPrS1 (Zemskova *et al.*, 2015) were cultured in RPMI supplemented with 2 mmol·L^−1^ GlutaMAX (Life Technologies, Rockville, MD, USA) and 10% FBS (BioAbChem, Ladson, SC, USA) at 37 °C under 5% CO_2_ as reported previously. The T‐ALL cell lines HSB‐2, DU.528, KOPT‐K1, CUTLL1, HPB‐ALL, and SUP‐T1 (Padi *et al.*, 2017) were cultured in RPMI 1640 supplemented with 2 mm GlutaMAX (Life Technologies) and 10% FBS (BioAbChem) at 37 °C under 5% CO_2_. LASCPC‐01 (ATCC) was grown in HITES media containing RPMI, 5% FBS, 10 nm hydrocortisone, 10 nm beta‐estradiol (Sigma‐aldrich, St. Louis, MO, USA), insulin–transferrin–selenium (Life Technologies), and GlutaMAX (Life Technologies). hPrEC and murine prostate epithelial tumor cells (mPrEC) cells were cultured as described previously (Song *et al.*, 2018). Phoenix‐Eco cells (a gift from J. Schatz, University of Miami Health System) and fibrosarcoma cell line FLYRD18/mCAT‐IRES‐Bleo (a gift from H.G. Wendel, Memorial Sloan Kettering Cancer Center) were cultured in DMEM + 2 mm glutamine + 10% FBS. All cell lines were maintained for no more than 6 months in culture and were routinely tested for Mycoplasma.

### 2.2 Organoid culture

The NEPC patient‐derived organoids OWCM‐155 were provided by H. Beltran and were cultured as previously described (Puca *et al.*, 2018). For murine organoids, cells were dissociated from the wild‐type (WT) mouse prostate and cultured as organoids as described (Drost *et al.*, 2016). All studies involving the use of animals were approved by and conducted in accordance with the guidelines of the Institutional Animal Care and Use Committees at the University of Arizona Cancer Center. Both NEPC and murine organoids were replenished with fresh media every 3–4 days during organoid growth. Dense cultures with organoids ranging in size from 200 to 500 μm were passaged weekly. Organoid cultures were biobanked using Bambanker (Gibco, Life Technologies) at −80 °C.

### 2.3 RNA extraction, qPCR, and gene expression analysis

Total RNA was isolated from cells using TRIzol reagent (Invitrogen, Waltham, MA, USA; Cat # 15596–018). One microgram total RNA was reverse‐transcribed by using i‐Script cDNA Synthesis System Kit (Bio‐Rad, Hercules, CA, USA; Cat # 1708891). To measure gene expression, real‐time PCR was performed using SsoAdvanced™ Universal SYBR® Green Supermix (Bio‐Rad; Cat # 1725271), following the manufacturer's protocol. Expression level of each transcript was quantified by using Bio‐Rad CFX96 Real‐Time PCR Detection System. Quantitative real‐time PCR (qPCR) assay was performed using the following primers (5′–3′):

**Table:** 

h‐H19‐F: GCACCTTGGACATCTGGAGT, h‐H19‐R: TTCTTTCCAGCCCTAGCTCA; primer for H19 was designed based on Ref Seq ID NR_002196.2; amplicon length: 171
h‐KLF4‐F: GGCACTACCGTAAACACACG, h‐KLF4‐R: CTGGCAGTGTGGGTCATATC; amplicon length: 140
h‐NANOG‐F: TTTGTGGGCCTGAAGAAAACT, h‐NANOG‐R: AGGGCTGTCCTGAATAAGCAG; amplicon length: 116
h‐OCT‐4‐F: TCGAGAACCGAGTGAGAGG, h‐OCT‐4‐R: GAACCACACTCGGACCACA; amplicon length: 125
h‐SOX2‐F: CCCTGTGGTTACCTCTTCCT, h‐SOX2‐R: AGTGCTGGGACATGTGAAGT; amplicon length: 136
h‐PIM1‐F: CGACATCAAGGACGAAAACATC, h‐PIM1‐R: ACTCTGGAGGGCTATACACTC; amplicon length: 137
h‐PIM2‐F: GAACATCCTGATAGACCTACGC, h‐PIM2‐R: CATGGTACTGGTGTCGAGAG; amplicon length: 142
h‐PIM3‐F: GACATCCCCTTCGAGCAG, h‐PIM3‐R: ATGGGCCGCAATCTGATC; amplicon length: 147
h‐c‐MYC‐F: AAACACAAACTTGAACAGCTAC, h‐c‐MYC‐R: ATTTGAGGCAGTTTACATTATGG; amplicon length: 188
h‐18S‐F: GTAACCCGTTGAACCCCATT, h‐18S‐R: CCATCCAATCGGTAGTAGCG; amplicon length: 151
m‐PIM1‐F: GATCATCAAGGGCCAAGTGT, m‐PIM1‐R: GATGGTTCCGGATTTCTTCA; amplicon length: 122
m‐OCT‐4‐F: TCAGGTTGGACTGGGCCTAGT, m‐OCT‐4‐R: GGAGGTTCCCTCTGAGTTGCTT; amplicon length: 100
m‐SOX2‐F: GGTTACCTCTTCCTCCCACTCCAG, m‐SOX2‐R: TCACATGTGCGACAGGGGCAG; amplicon length: 193
m‐KLF4‐F: CCAAAGAGGGGAAGAAGGTCG, m‐KLF4‐R: GTGCCTGGTCAGTTCATCGG; amplicon length: 198
m‐HPRT‐F: AAGCTTGCTGGTGAAAAGGA, m‐HPRT‐R: TTGCGCTCATCTTAGGCTTT; amplicon length: 186

### 2.4 Immunoblotting

As previously described (Padi *et al.*, 2017), at the end of each experiment, cells were lysed in RIPA buffer (Cell Signaling Technology, Danvers, MA, USA, Cat # 9806S) with complete protease/phosphatase inhibitor cocktail (Cell Signaling Technology, Cat # 5872S). The protein concentration was determined by Bio‐Rad DC Protein Assay (Bio‐Rad). Western blots (WBs) were performed as described previously (Song *et al.*, 2018). The levels of PIM1 kinase (relative to ACTIN) and p‐IRS1 (S1101) proteins (relative to IRS1) were quantified and normalized to their respective control samples using imagej (NIH, https://imagej.nih.gov/ij) software.

### 2.5 Antibodies and reagents

Primary antibodies used for western blotting included anti‐PIM1 (Cell Signaling Technology, Cat # 2907), anti‐PIM2 (Cell Signaling Technology, Cat # 4730), anti‐PIM3 (Cell Signaling Technology, Cat # 4165), anti‐pIRS1‐S1101 (Cell Signaling Technology, Cat # S1101 Cat # 2385), anti‐insulin receptor substrate 1 (IRS1; Cell Signaling Technology, Cat # 06‐248), anti‐HA (Cell Signaling Technology, Cat # 14031), anti‐SOX2 [(E‐4) sc‐365823; Santa Cruz Biotech, Santa Cruz, CA, USA], anti‐OCT‐4 [(C‐10) sc‐5279; Santa Cruz Biotech], and anti‐NANOG [(5A10) sc‐134218; Santa Cruz Biotech]. HRP‐conjugated anti‐β‐actin (Cat # A3854) was purchased from Sigma‐Aldrich. HRP‐linked mouse IgG (Cat # NA931V) and rabbit IgG (Cat # NAV934V) were purchased from GE Healthcare Life Sciences (Princeton, NJ, USA).

Doxycycline (Dox) hydrochloride (Cat # D3447) was purchased from Sigma‐Aldrich. AZD1208 (Cat # A13203) was purchased from Adooq Bioscience (Irvine, CA, USA). Enza (MDV3100; Cat # S1250) was purchased from Selleckchem (Houston, TX, USA). PIM447 was a gift from Novartis (Basel, Switzerland).

### 2.6 Plasmids

Knockdown of human H19 was performed using the lentiviral plasmids pLenti‐siH19‐GFP (Abm, Richmond, British Columbia, Canada, Cat # i009382) and pLenti‐scrambled siRNA‐GFP (Abm, Cat # LV015‐G) as a control. These siH19 plasmids allow for direct nonviral plasmid transfection for immediate expression (siH19) and also package into lentiviral particles for high‐efficiency transduction and stably integrated expression (shH19). Overexpression of human H19 was performed using pLenti‐GIII‐CMV‐H19‐GFP‐2A‐Puro (Abm, Cat # LV178008). The PIM1‐expressing constructs and its K67M kinase‐dead (KiD) mutants were described previously (Cen *et al.*, 2010). All the PIM1, PIM2, and PIM3 overexpression plasmids and siRNA to PIM1 were performed as previously described (Padi *et al.*, 2017; Song *et al.*, 2018). Transient transfection of siRNA and cDNA was performed using Lipofectamine 3000 (Invitrogen) and Xfect transfection reagent (Clontech, Takara Bio USA Inc., Mountain View, CA, USA). The SUP‐T1 cells were engineered (SUP‐T1E) using a fibrosarcoma cell line FLYRD18/mCAT‐IRES‐B (Ngo *et al.*, 2006) and infected with MigR1 and MigPIM1 following established procedures (Peters *et al.*, 2016).

### 2.7 Cell viability assay

LNCaP and PC3 cell lines were seeded into 96‐well plates at a density of 5000 cells per well and allowed to grow under desired conditions. As described previously (Padi *et al.*, 2017), at the end of each experiment, cell viability was measured using XTT cell proliferation assay (Trevigen, Gaithersburg, MD, USA; Cat # 4891‐025‐K) following the manufacturer's protocol. Briefly, the XTT reagent was added to cell culture (1 : 2 dilution) and incubated for 4 h at 37 °C and 5% CO_2_. The absorbance of the colored formazan product was measured at 450 nm.

### 2.8 Organoid growth assay

Organoids were dissociated with TrypLE (Invitrogen) into tiny cell clusters, plated (5000 cell clusters per well), and treated under desired conditions for 4–6 days. A real‐time imaging system (IncuCyte™; Essen Bioscience, Sartorius, Ann Arbor, MI, USA) was used to measure organoid growth. Images were captured every 12 h, and results were plotted as the percent average organoid growth vs time.

### 2.9 Luciferase assay

PC3 cells stably overexpressing Dox‐inducible PIM1 (PC3 Tripz‐PIM1) were plated in 96‐well plates (2 × 10^4^ cells per well) and transfected with a OCT‐4 (proximal and distal enhancer) luciferase reporters (Addgene, Watertown, MA, USA) or Renilla (Promega, Madison, WI, USA) using Lipofectamine (Invitrogen). The plasmid sequences for OCT‐4 distal and proximal enhancers were pGL3‐human OCT‐4 DE‐SV40‐Luc and pGL3‐human OCT‐4 PE‐SV40‐Luc and were a gift from J. Hanna (Addgene plasmid # 52414 and 52415, respectively) (Gafni *et al.*, 2013). The total plasmid DNA used was normalized to 0.5μg per well by the addition of Renilla. At 24 h after transfection, luciferase activities were measured using a Dual‐Luciferase Reporter Assay System (Promega) and a GloMax 96‐well microplate luminometer (Promega). OCT‐4 (proximal and distal promoter) luciferase activities were corrected by the corresponding Renilla luciferase activities. Results are expressed in arbitrary light units.

### 2.10 Lentiviral production and transduction

Lentiviral particle production and infection were performed as described previously (Tiscornia *et al.*, 2006). For infection of adherent PCa cells, 10^6^ cells per well were seeded in six‐well plates and infected with concentrated lentiviral particles 1 day after seeding. For lentiviral transduction, organoids were preincubated for at least 48 h with regular organoid media supplemented with Wnt‐3a and Rho kinase inhibitor (Karthaus *et al.*, 2014). After 48 h, organoids were dissociated with TrypLE and spinoculated with lentiviral particles along with polybrene (8 μm final concentration) at 600 g for 1 h at 32 °C. After incubating organoid cells at 37 °C for 3 h, the cells were replated in Matrigel in ENR media without lentivirus and allowed to grow for several days.

### 2.11 Bisulfite sequencing

Genomic DNA from informative samples were treated with bisulfite (EpiTect Plus DNA Bisulfite Kit; Qiagen, Hilden, Germany; Cat No./ID: 59124) to convert unmethylated cytosines to uracils, whereas methylated cytosines are unaffected according to the manufacturer's protocol. Bisulfite‐treated DNA was subsequently amplified using the forward 5′‐TGGGTATTTTTGGAGGTTTTTTT‐3′ and reverse 5′‐TCCCATAAATATCCTATTCCCA 3′ primers.

### 2.12 Fluorescent‐activated cell sorting

Cells were resuspended in 10% FBS/PBS to reach a concentration of 10^7^ cells per milliliter. Twenty microliters of the cell suspension was stained with various antibodies diluted in 10% FBS/PBS for 1 h. Subsequently, cells were washed with 2% FBS/PBS and resuspended in 10% FBS/PBS for flow cytometry analysis (FACS). Antibodies used for our FACS analyses include APC anti‐human CD24 antibody (Cat # 311117; Biolegend, San Diego, CA, USA), PE anti‐human CD29 antibody (Cat # 303003; Biolegend), APC anti‐human CD133 (Cat # 17‐1338‐41; eBioscience, San Diego, CA, USA), and APC anti‐human CD49b (integrin alpha‐2; Cat # 17‐0500‐41; eBioscience).

### 2.13 Affymetrix gene chip expression analysis

The microarray was performed as described previously (Padi *et al.*, 2017). Total RNA was extracted from six T‐ALL cell lines using the RNeasy kit following manufacturer's instructions (Qiagen, Cat #74104). The Genomics Facility Core at University of Arizona Cancer Center performed quality control using the Agilent Bioanalyzer 2100 to confirm all RNA samples had RNA integrity numbers greater than seven and to quantitate the concentration. From the RNA, the Genomics Core produced labeled a DNA target using the WT PLUS Reagent Kit and hybridized it to the Affymetrix® HTA 2.0 Array (Santa Clara, CA, USA) overnight according to the manufacturer's instructions. Arrays were washed and scanned with the GeneChip Hybridization, Wash, and Stain Kit and an Affymetrix® Scanner 3000 (Santa Clara, CA, USA) following the manufacturer's instructions. The Affymetrix® transcriptome analysis console v3.0 software (Affymetrix) was used to analyze resulting data file to identify differentially expressed genes between PIM inhibitor (PIM‐i)‐sensitive cells (HSB‐2, DU.528, and KOPT‐K1) and PIM‐i‐insensitive cells (CUTLL1, HPB‐ALL, and SUP‐T1) and generated a scatter plot of differentially expressed genes with the following criteria: fold change (linear) < −2 or fold change (linear) > +2, and ANOVA *P* value (condition pair) < 0.05.

### 2.14 Statistics

Values reported and shown in graphical displays are the mean ± standard deviation or standard error of the mean, as indicated. Comparisons of mean expression across groups were made using an unpaired two‐tailed Student's *t*‐test. For all comparisons, *P* values < 0.05 were considered statistically significant.

## 3 Results

### 3.1 PIM protein kinase regulates the level of the lncRNA H19

**Fig. 1:**
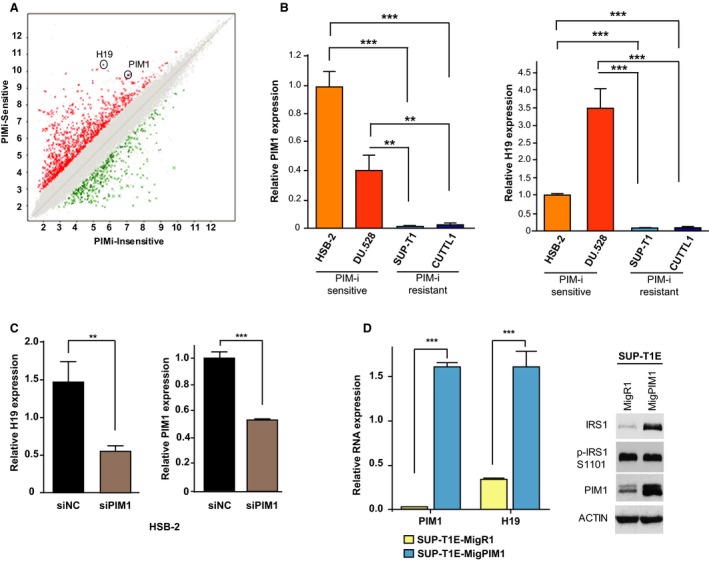
PIM induces H19 expression in T‐ALL cell lines. (A) Scatter plot of genes that are significantly different [fold change (linear) < −2 or > + 2 and ANOVA *P* value (condition pair) < 0.05) comparing the RNA levels of PIM‐i‐sensitive cells with PIM‐i‐resistant T‐ALL cell lines. H19 and PIM1 RNA are circled. Expression levels are visualized as color‐coded with red indicating higher levels and green indicating lower levels of gene expression. (B) Relative PIM1 RNA expression in HSB‐2, DU.528, SUP‐T1, and CUTLL1. (C) Relative RNA expression of H19 in HSB‐2, DU.528, SUP‐T1, and CUTLL1. (D) Relative RNA expression of H19 and PIM1 in HSB‐2 cells transfected with an siRNA to a negative control (siNC as control) or PIM1 (si‐PIM1). (E) Relative RNA expression of PIM1 and H19 in SUP‐T1 cells that were engineered (SUP‐T1E) for infection with control vector MigR1 (SUP‐T1E MigR1) or PIM1‐overexpressing vector MigPIM1 (SUP‐T1E MigPIM1). The WB was performed with the indicated antibodies. ACTIN was used as loading control. (B–E) Relative RNA expression was normalized to 18S RNA levels. Values are mean ± SEM; *n* = 3; **P < 0.01, and ***P < 0.001.

We have chosen to examine two model systems to understand the mechanism by which the family of PIM kinases regulates tumor growth. T‐ALL and PCa have been shown to be driven by increased PIM levels, and small molecule inhibitors of PIM decrease the growth of these tumor types. T‐ALL cell lines can be divided into the early progenitor types (ETP‐ALL), HSB‐2, KOPT‐K1, and DU.528, containing elevated levels of PIM kinase, and those that are more mature, SUP‐T1, HPB‐ALL and CUTLL1, and express lower levels of this protein kinase (Padi *et al.*, 2017). ETP‐ALL cells are blocked in their growth by PIM‐i, while the more mature T‐ALLs, SUP‐T1, and CUTLL1 are not (Padi *et al.*, 2017). To understand the genes or pathways that might be involved in the sensitivity of HSB‐2, KOPT‐K1, and DU.528 and the resistance of SUP‐T1, HPB‐ALL, and CUTLL1 to PIM‐i, microarray profiling was carried out on these six T‐ALL cell lines (Fig. 1A, Table S1). Among the genes most elevated in ETP‐ALL cells whose growth is driven by PIM and sensitive to PIM‐i therapy, the lncRNA H19 was identified (Fig. 1A). Using qPCR, we confirmed that H19 is highly expressed in the PIM‐driven PIM‐i‐sensitive HSB‐2 and DU.528 but at much lower levels in the PIM‐i‐insensitive SUP‐T1 and CUTLL1 cells (Fig. 1B,C, Fig. S1). KD of PIM1 levels in HSB‐2 with siRNA led to a significant decrease in cellular H19 level (Fig. 1D), demonstrating that PIM1 regulates H19 in these ETP‐ALL cells. Conversely, PIM1 overexpression in the PIM‐i‐resistant cell line SUP‐T1 induced increases in H19 levels (Fig. 1E). The PIM1 overexpression in SUP‐T1E cells was confirmed by measuring p‐IRS1 (S‐1101; Fig. 1E). IRS1 is a known PIM kinase substrate, and its phosphorylation is regulated by PIM kinase activity (Song *et al.*, 2016).

A possible mechanism by which PIM regulates H19 levels is by modulation of the H19 DMR methylation. There is a striking difference in the methylation state of H19 between PCa and benign prostate hyperplasia (BPH). Eight percent of the CpGs in the DMRs and in the ICRs are methylated in BPH, while only 41% of CpGs are methylated in PCa (Paradowska *et al.*, 2009). Bisulfite sequencing demonstrates that while the DMR is methylated in 100% of the SUP‐T1 clones, PIM1‐overexpressing SUP‐T1 cells demonstrated a demethylation of the DMR in 30% of the clones analyzed, reflecting the loss of imprinting of H19 (Fig. S2A,B). This result suggests a potential mechanism for the suppression of H19 expression in SUP‐T1 cells and its potential regulation by PIM1 kinase.

**Fig. 2:**
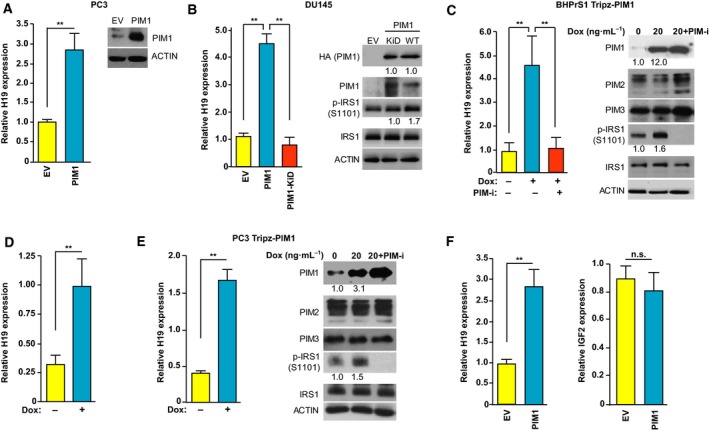
PIM1 induces H19 expression in PCa cell lines. (A) Relative H19 RNA expression in PC3 cells transfected with EV as control or FUCRW‐PIM1 (PIM1). PIM1 protein expression was validated by WB. (B) Relative H19 RNA expression in DU145 cells transfected with pcDNA3 vector encoding either PIM1 (PIM1) or a mutated PIM1‐KiD. DU145 (EV) cells were used as control. Respective transfections and PIM1 overexpression were validated by WB. (C) Relative H19 RNA expression in BHPrS1 cells stably expressing Tripz vector encoding Dox‐inducible PIM1 (BHPrS1 Tripz‐PIM1). H19 expression level was measured by qPCR after Dox treatment (20 ng·mL^−1^, 24 h) with or without overnight incubation with PIM‐i (AZD1208, 3 μm). The WB was performed with the indicated antibodies. (D) Relative H19 RNA expression in normal human prostate‐derived epithelial cells, hPrEC cells stably expressing Tripz vector encoding Dox‐inducible PIM1. H19 expression level was measured after Dox treatment (20 ng·mL^−1^, 24 h) by qPCR. (E) Relative H19 RNA expression in PC3 cells stably expressing Tripz vector encoding Dox‐inducible PIM1 (PC3 Tripz‐PIM1). H19 expression level was measured after Dox treatment (20 ng·mL^−1^, 24 h) by qPCR. WB was performed with the indicated antibodies. Inhibitor treatments are the same as in C. (F) Relative H19 and IGF2 RNA expression in PC3 cells transfected with EV as control or FUCRW‐PIM1 (PIM1). (A–C, E) For WB: ACTIN was used as loading control. Numerical values shown under the blot are derived as described in Materials and Methods and calculated relative to the controls. (A–F) Relative RNA expression was normalized to 18S RNA levels. Values are mean ± SEM; *n* = 3; n.s., not significant, **P < 0.01.

Since PIM1 kinases have been shown to drive the growth of PCa, we sought to address whether the PIM1 regulation of H19 levels was also present in this tumor type. Transient overexpression of PIM1 in human PCa cell lines PC3 and DU145 elevated H19 expression (Fig. 2A,B). To examine whether the kinase activity of this enzyme was needed, DU145 cells were transfected with PIM1 (WT) or a K67M KiD mutant (Cen *et al.*, 2010) (Fig. 2B). WT‐PIM1, but not its KiD mutant, induced increases in H19 levels (Fig. 2B). Similarly, in human prostate stromal cell line BHPrS1 containing Dox‐inducible PIM1 construct (Zemskova *et al.*, 2015), PIM1 induction resulted in increases in H19 levels, which were blocked by 24 h of PIM‐i (AZD1208, 3 µm) treatment (Fig. 2C). The effect of Dox induction and PIM‐i treatment was confirmed by p‐IRS1 phosphorylation (Fig. 2C). Similarly, PIM1‐mediated H19 induction was also observed in normal human prostate‐derived epithelial cells, hPrEC (Fig. 2D), or prostate tumor cell line PC3 (Fig. 2E). To check whether this H19 induction by PIM kinase is isoform‐specific, we transduced PC3 cells with PIM1, PIM2, or PIM3 and measured the H19 levels. Each of the PIM kinase isoforms induced similar H19 level increases (Fig. S3A), indicating that each isoform was capable of regulating this lncRNA.

H19 and IGF2 are expressed from the same genetic locus (Gabory *et al.*, 2010). We observed no changes in IGF2 RNA expression when PIM1 kinase was transfected in PC3 cells (Fig. 2F). Since H19 has been shown to be directly activated by c‐MYC (Barsyte‐Lovejoy *et al.*, 2006), the increase in H19 upon PIM overexpression could be mediated by changes in c‐MYC levels. It has been shown that the PIM1/PIM2 kinases synergize with c‐MYC to induce tumorigenesis. It has been found that overexpressing PIM1 kinase decreased phosphorylation of Thr58 and enhanced phosphorylation at S62, whereas PIM2 caused S329 phosphorylation on c‐MYC. These phosphorylation events caused by PIM1/PIM2 lead to increased protein stability and enhanced transcriptional activity of c‐MYC (Kim *et al.*, 2010; Zhang *et al.*, 2008). However, in our model system, overexpression of c‐MYC in DU145 cells did not induce H19, suggesting that PIM regulates H19 independently of c‐MYC function (Fig. S4). Taken together, these data demonstrate that the lncRNA H19 expression is regulated by the PIM kinases in both hematopoietic and solid tumor types.

### 3.2 PIM overexpression is associated with a stem cell signature

**Fig. 3:**
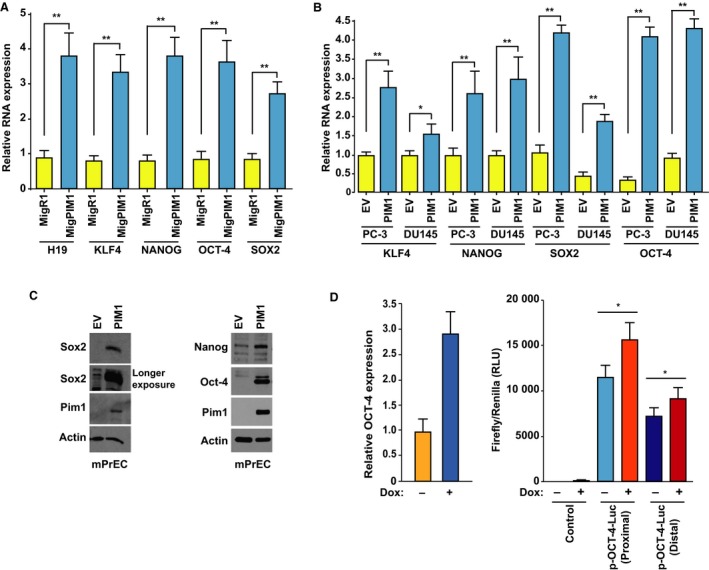
PIM1 regulates stem cells genes in T‐ALL and PCa cell lines. (A) Relative RNA expression of H19, KLF4, NANOG, OCT‐4, and SOX2 in SUP‐T1E cells transfected with control MigR1 vector (MigR1) or PIM1‐overexpressing vector (MigPIM1). (B) Relative RNA expression of KLF4, NANOG, SOX2, and OCT‐4 in PC3 and DU145 cells transduced with EV or PIM1 vector (PIM1). (C) WB using the identified antibodies on mPrEC cells in which Trp53 and Rb1 had been knocked out, and stably transduced with EV plasmid or FUCRW‐PIM1 (PIM1) plasmid. (D) Relative OCT‐4 RNA expression in Dox‐inducible PC3‐Tripz‐PIM1 after Dox treatment (20 ng·mL^−1^, 48 h). Relative Firefly/Renilla luciferase activity measurement after Dox treatment (20 ng·mL^−1^, 48 h) in PC3‐Tripz‐PIM1 cells transfected with luciferase reporter plasmid (p‐OCT‐4‐Luc) controlled by OCT‐4 proximal (Proximal) and distal (Distal) enhancers. Untransfected PC3‐Tripz‐PIM1 cells were used as control. (A, B, D) Relative RNA expression was normalized to 18S RNA levels. Values are mean ± SEM; *n* = 3; *P < 0.05, **P < 0.01.

H19 expression has been shown to positively correlate with the level of stem cell genes and pluripotency factors in various tumor types (Bauderlique‐Le Roy *et al.*, 2015; Li *et al.*, 2016; Peng *et al.*, 2017; Zeira *et al.*, 2015). Comparison of the PIM‐i‐resistant (SUP‐T1 and CUTLL1) and PIM‐i‐sensitive (HSB‐2 and DU.528) T‐ALL cell lines revealed significantly elevated SOX2 expression in sensitive vs resistant T‐ALL cells (Fig. S1). PIM1 overexpression in both T‐ALL cell line—SUP‐T1—and PCa cell lines—PC3 and DU145—was able to induce the expression of stem cell factors OCT‐4, SOX2, NANOG, and KLF4 (Fig. 3A,B). At the protein level, PIM1 overexpression in murine PCa cells mPrEC (Song *et al.*, 2018) augmented the level of the stem cell genes Sox2, NANOG, and Oct‐4 (Fig. 3C). The overexpression of PIM2 and PIM3 in PC3 cells also led to increases in NANOG and OCT‐4 gene expression (Fig. S3A). Using a luciferase reporter controlled by either the OCT‐4 proximal or distal enhancers, increasing PIM1 levels in PC3 cells showed increased OCT‐4 transcription and promoter activity when PIM1 was induced (Fig. 3D). To confirm that the PIM1‐induced stem cell signature is associated with a stemness state, we analyzed PIM1‐overexpressing cells for the enrichment of stem cell surface markers (CD24 (Weng *et al.*, 2019), CD29 (Lawson *et al.*, 2010; Vassilopoulos *et al.*, 2014), and CD49B (Erb *et al.*, 2018; Lawson *et al.*, 2010). We demonstrate that PIM1 overexpression in PC3 cells induces stem cell surface markers CD29, CD49b, and CD24, suggesting that increased PIM1 expression is capable of inducing a stem cell‐like surface phenotype through in part regulating these gene changes (Fig. S3B).

**Fig. 4:**
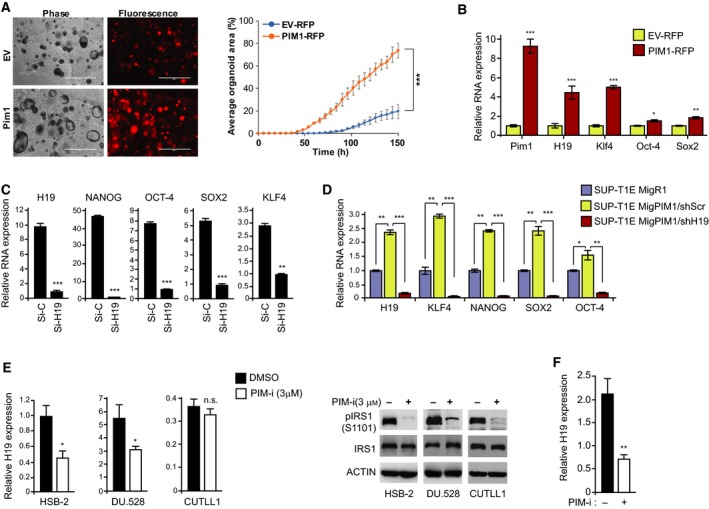
PIM1 controls H19 expression and regulates tumor growth. (A) Mouse prostate organoid cells transduced with EV (EV‐RFP) or FUCRW‐PIM1 plasmid (PIM1‐RFP; 5000/well) were plated for organoid growth analysis. Representative images of the growth analysis (left panel), scale bar: 1000 μm. Quantification of the organoid growth (right panel). (B) Relative RNA levels of Pim1, H19, Klf4, Oct‐4, and Sox2 in mouse prostate organoids described in A. Relative RNA expression was normalized to Hprt RNA. (C) Relative RNA expression of NANOG, OCT‐4, SOX2, and KLF4 in PC3‐LN4 cells transfected with a control siRNA (si‐C) and siRNA to KD H19 (si‐H19). Relative RNA expression levels were normalized to ACTIN RNA. (D) Relative RNA expression of H19, KLF4, NANOG, OCT‐4, and SOX2 in SUP‐T1E cells transfected with control MigR1 vector (MigR1) or PIM1‐overexpressing vector (MigPIM1). Mig PIM1 cells were further transduced with shScr and shH19. (E) Relative H19 RNA expression in HSB‐2, DU.528, and CUTLL1 after PIM‐i treatment (AZD1208, 3 μm, 24 h). DMSO was used for control treatment. Relative RNA expression levels were normalized to ACTIN RNA. WB of these cells with indicated antibodies for validation of PIM1 inhibition. (F) Relative H19 RNA expression in LNCaP cells treated with PIM‐i (PIM447, 1 μm, 24 h). DMSO was used for control treatment. Relative RNA expression was normalized to 18S RNA unless specified otherwise. Values are mean ± SEM; *n* = 3; n.s., not significant, *P < 0.05, **P < 0.01, ***P < 0.001.

To evaluate the effect of PIM1 overexpression on growth of normal stem cells, we isolated WT mouse prostate organoids that have been shown to possess stem cell‐like characteristics (Karthaus *et al.*, 2014). PIM1 overexpression in these organoids caused a significant increase in proliferation of these cells (Fig. 4A; PIM1‐RFP vs EV‐RFP, *P* < 0.001). As in PCa cell lines, overexpressing PIM1 in mouse organoids induced increases in H19 and the stem cell genes Klf4, Oct‐4, and Sox2 (Fig. 4B). Importantly, in PC3‐LN4 cells, a metastatic variant of PC3 cells that expresses elevated levels of the PIM kinases (Song *et al.*, 2018), H19 KD repressed the expression of NANOG, OCT‐4, SOX2, and KLF4 (Fig. 4C).

To validate that the PIM1 increases in stem cell genes are mediated by H19, we knocked down H19 in PIM1‐overexpressing SUP‐T1E cells using either lentivirus encoding scrambled shRNA (shScr) as a control or shH19 (Fig. 4D). Our results indicated that the KD of H19 abrogated the PIM1‐induced stem cell gene expression. Thus, these experiments help to establish a novel signaling cascade connecting PIM1, H19, and stem cell genes, suggesting that the PIM1 induction of stem cell genes may occur with the activity of H19.

### 3.3 PIM‐i treatment reduces H19 expression and inhibits cell growth

To investigate the effect of pharmacological inhibition of PIM kinases on the levels of H19 in T‐ALL cell lines, HSB‐2, DU.528, and CUTLL1 were treated with the pan‐PIM‐i AZ1208 (3 µm, 24 h). This treatment reduced H19 levels in the two ETP‐ALL cell lines, HSB‐2 and DU.528, by ~ 60%, while it had no effect on H19 levels in the more mature and kinase inhibitor‐resistant CUTLL1 cells (Fig. 4E). Similarly, treatment of LNCaP cells (Fig. 4F) with PIM‐i decreased H19 levels. Similar results were obtained in NEPC cell line LASCPC‐01 where PIM447, another small molecule pan‐PIM‐i, significantly decreased H19, NANOG, OCT‐4, and SOX2 expression in a dose‐dependent manner (Fig. S5). Additionally, we observed that overexpression of PIM1 partially sensitized PIM‐i‐resistant SUP‐T1E cells to PIM‐i treatment (Fig. S6). These data demonstrate that PIM1 regulates H19 levels in PIM‐dependent but not in PIM‐i‐insensitive cell lines.

**Fig. 5:**
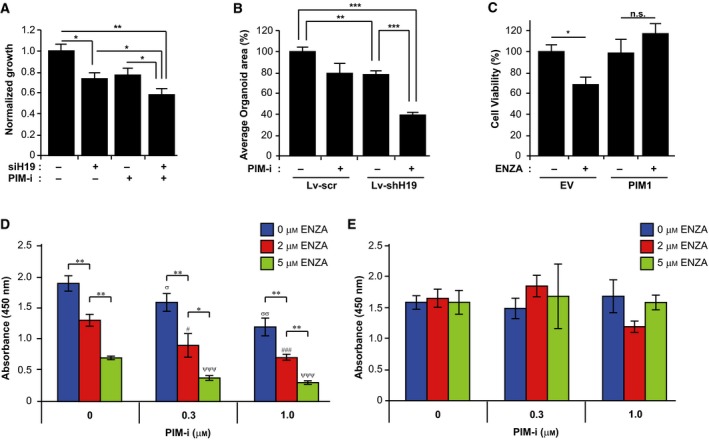
PIM‐i represses growth in combination with inhibition of H19 and sensitizes LNCaP cells to Enza. (A) Cell viability measured by XTT assay of PC3 cells treated with si‐H19 and PIM‐i (PIM‐447, 0.5 μm, 72 h). DMSO was used for the control treatment. Cell viability is represented as normalized growth (DMSO = 1). (B) Bar graphs showing growth response measured as percent average organoid area of OWCM‐155 NEPC organoids with and without KD of H19 (Lv‐shH19 vs Lv‐scr) in combination with PIM1‐i (PIM447, 3 μm, 4 days). DMSO was used for control treatment. (C) Percent cell viability of LNCaP cells stably expressing EV or FUCRW‐PIM1 plasmid (PIM1), treated with Enza (MDV3100, 10 μm, 48 h). DMSO treatment was used as control. (D) Cell viability of LNCaP cells stably expressing EV (LNCaP‐EV) treated with increasing concentrations of either Enza (MDV3100, 2 and 5 μm) or PIM‐i (PIM447, 0.3 and 1 μm, 72 h) and combination, measured by XTT assay. DMSO was used to treat cells when no drug was added (0 μm). Values are mean ± SEM; *n* = 3; σ represents *P* values < 0.05 as compared to DMSO treatment. # represents *P* values < 0.05 as compared to 2 µm Enza. Ψ represents *P* values < 0.05 as compared to 5 µm Enza. *P < 0.05, **P < 0.01, (E) Cell viability of LNCaP cells stably overexpressing H19 (LNCaP‐H19) treated with Enza or PIM‐i and combination, as stated in D. XTT assay was carried out 72 h after the start of drug treatment, and absorbance was measured at 450 nm. Results from cell viability in D and E were represented as absorbance at 450 nm. Values are mean ± SEM; *n* = 3; n.s., not significant, *P < 0.05, **P < 0.01, ***P < 0.001.

To analyze whether the inhibition of PIM and the subsequent decrease in H19 affected cell growth in PCa, we performed XTT assay on PC3 cells with H19 KD and treated or not with a PIM‐i. The combination of si‐H19 and PIM‐i significantly inhibited cell growth (Fig. 5A), while individual treatments had modest effects. NEPC is a variant of PCa that is clinically unresponsive to chemotherapy treatments. To test this combination treatment in this tumor type, we utilized NEPC patient‐derived organoids OWCM‐155 (Puca *et al.*, 2018), which have high levels of H19 (Singh et al., manuscript in preparation). When compared to androgen‐responsive PCa cell line LNCaP, these organoids have elevated PIM kinase expression (Fig. S7). Stable KD of H19 in these organoids (Fig. S8) results in a significant growth reduction (Fig. 5B). When H19 KD and PIM‐i treatment are combined, there is an even further decrease in the NEPC organoid growth (Fig. 5B). These results demonstrate that kinase inhibition and decreases in this lncRNA can function together to inhibit PCa growth.

### 3.4 H19 blocks the ability of PIM‐i to sensitize prostate cancer cells to hormone blockade

Patients diagnosed with advanced PCa initially respond to androgen deprivation therapy (ADT) but ultimately develop hormone‐refractory disease that leads to death. Recent evidence suggests that increased expression of the reprogramming stem cell transcription factor SOX2 makes PCa cells resistant to hormone blockade by the antiandrogen Enza (Mu *et al.*, 2017). To investigate the role of PIM kinases in ADT, we analyzed the effect of Enza treatment on PIM1‐overexpressing androgen‐responsive PCa cell line LNCaP cells. Interestingly, we observed that while Enza treatment (48 h, 10 μm) was cytotoxic to the empty vector (EV)‐transduced control LNCaP cells (EV), overexpression of PIM1 caused resistance to Enza treatment (Fig. 5C). This result is consistent with our finding that PIM kinase activity stimulates increases in SOX2 (Fig. 3C). To explore this further, we examined the growth of LNCaP with control vector transduction (LNCaP‐EV) and H19 overexpression (LNCaP‐H19; Fig. S9), followed by treatment with increasing concentrations of PIM447 with or without Enza (2, 5 µm) treatment for 72 h (Fig. 5D,E). The PIM‐i treatment significantly sensitized the control LNCaP cells to growth inhibition by Enza (Fig. 5D). Notably, ComboSyn analysis (Chou, 2010) demonstrated that the PIM447 and Enza combination was highly synergistic in killing LNCaP cells. As shown in Fig. S9, the combination index values were < 1 for multiple PIM447 and Enza combination doses. Overexpression of H19 in LNCaP cells blocked the effect of PIM kinase inhibitor treatment (Fig. 5E), suggesting that the PIM‐mediated control of H19 levels modulates Enza sensitivity in LNCaP cells.

## 4 Discussion

Our results identify a unique signaling cascade that regulates the transcription of stem cell genes in both T‐ALL and PCa. PIM1 kinase induces the expression of lncRNA H19, which in turn stimulates the transcription of the stem cell genes SOX2, NANOG, and OCT‐4. We found that the expression of H19 is significantly higher in the T‐ALL cell lines with high levels of PIM1 kinase and sensitivity to PIM‐i (HSB‐2, DU.528, and KOPT‐K1) as compared to those with low PIM1 levels and insensitivity to these kinase inhibitors (CUTLL1, HPB‐ALL, and SUP‐T1). Since PIM kinases (Aksoy *et al.*, 2007; Jimenez‐Garcia *et al.*, 2017; Xie and Bayakhmetov, 2016) and H19 (Peng *et al.*, 2017; Zimta *et al.*, 2019) have been both shown to be involved in the maintenance of stemness in solid tumors and hematopoietic malignancies, we decided to investigate whether there is a link between the two in the regulation of stem cell genes. We further demonstrated that PIM‐i decreased H19 levels in PIM‐i‐sensitive cell lines (HSB‐2 and DU.528) but has no effect on H19 levels in the PIM‐i‐insensitive cell line CUTLL1. Overexpression of PIM1 in SUP‐T1 cells increased stem cell gene transcription through H19 and restored partial sensitivity to PIM‐i. Further, we identified that the stem cell genes SOX2, KLF4, NANOG, and OCT‐4 were induced as a result of H19 elevation. These findings were corroborated in PCa cells, indicating the PIM regulation of H19 and induction of stem cell genes in both solid and hematopoietic tumors.

Further analysis of the microarray data comparing the transcriptomes of PIM‐i‐sensitive vs PIM‐i‐insensitive cells revealed enrichment of several pathways important for stem cell progression and maintenance (Table S2) in the PIM‐i‐sensitive cell lines. Studies have shown the involvement of these pathways in maintenance of pluripotent stem cells both in solid tumors and in hematopoietic malignancies. Specific pathways of interest could be chemokine signaling pathway (*P* value = 0.00019) (Jiang *et al.*, 2017), VEGFA/ VEGFR2 pathway (*P* value = 0.0002) (O'Donnell *et al.*, 2016), and PI3K‐Akt signaling pathway (*P* value = 0.045) (Madsen *et al.*, 2019) that are enriched in the PIM‐i‐sensitive cell lines and could be presumably more important in understanding the PIM‐specific mechanism. Further experiments are needed to examine the role of PIM and H19 in regulating a broad segment of the genes regulating this stem cell phenotype.

The mechanism by which H19 mediates stemness in liquid and solid tumors (Jiang *et al.*, 2016; Peng *et al.*, 2017; Ren *et al.*, 2018; Sasaki *et al.*, 2018; Zhou *et al.*, 2019) is likely complex. Various studies have pointed out the ‘sponge effect’ of H19 in which this lncRNA decreases the bioavailability of miRNAs such as Let7, leading to an increase in their target gene expression (RAS, MYC, HMGA2, STAT3, and IL‐10) (Peng *et al.*, 2017; Zhou *et al.*, 2017). H19 forms a double‐negative circuit with let‐7 and LIN28 in breast cancer cells, wherein H19 sponges the let7 miRNA that consequently releases and promotes the LIN28 expression. This increase in LIN28 expression further potentiates H19 expression, leading to the feedback circuit that acts to maintain the stem cell state in breast cancer (Peng *et al.*, 2017). The LIN28/let7 has also been associated with increasing the stem cell gene expression in oral squamous cell carcinoma (Chien *et al.*, 2015). The suppression of Let7 by LIN28 derepresses its target mRNAs—ARID3B and HMGA2, which then is thought to increase the transcription of stem cell genes OCT‐4 and SOX2, through promoter binding. However, further studies are needed to investigate the effect of H19 on this double‐negative feedback loop in the context of PIM1‐overexpressing PCa or T‐ALL cells.

Alternatively, H19 may function to increase stem cell gene levels through epigenetic mechanisms. H19 has been documented to bind to *S*‐adenosylhomocysteine hydrolase protein inducing genome‐wide methylation changes by indirectly regulating *S*‐adenosylmethionine‐dependent methyltransferases (Zhou *et al.*, 2015). This H19‐driven methylation could be sufficient to increase the transcription of stem cell genes. It has been demonstrated by our laboratory (Singh et al., manuscript in preparation) and others that H19 physically interacts with the proteins in the polycomb repressor complex 2 (PRC2) complex, for example, enhancer of zeste homolog 2, and increases histone H3K27me3 modifications (Fazi *et al.*, 2018). It is possible that similar to the lncRNA HOTAIR (Tsai *et al.*, 2010), H19 could bind both the PRC2 complex and lysine demethylases to mutually activate and repress gene transcription inducing a ‘stem‐like’ state.

The PIM kinases may function to elevate H19 levels by regulating both transcription factor binding and DNA methylation. The promoter region of H19 contains transcription factor binding sites for HIF1 (Wu *et al.*, 2017), androgen receptor (AR; putative), E2F1 (Berteaux *et al.*, 2005), and OCT‐4/SOX2 (Zimmerman *et al.*, 2013). In hypoxia, PIM kinases are elevated and increase HIF1 activity (Casillas *et al.*, 2018), thus having the potential to activate H19 transcription. H19 levels have been shown to be hormonally regulated and are inhibited by dihydrotestosterone in androgen‐responsive cells (Berteaux *et al.*, 2004), thus pointing to an inverse relation between AR and H19. PIM1 can regulate AR levels by phosphorylation, leading to degradation of this protein (Linn *et al.*, 2012) potentially removing a negative regulator controlling the transcription of this lncRNA.

All the PIM isoforms phosphorylate p27 protein leading to its degradation, stimulating the cell cycle (Morishita *et al.*, 2008), which can indirectly inhibit RB activity. When RB1 activity is lost, E2F1 could bind to the H19 promoter and stimulate increases in its transcription (Berteaux *et al.*, 2005). In turn, H19 affects RB1 phosphorylation by regulating the expression of CDK4 and CCND1 genes (Ohtsuka *et al.*, 2016) that can act to further increase E2F1 activity. In murine ES cells, OCT‐4 and SOX2 have been shown to cooperatively bind the ICR of the Igf2/H19 locus, resulting in a hypomethylated state, which stimulates increases in this lncRNA (Zimmerman *et al.*, 2013). Mass spectrometry on PIM1‐treated ES cell samples revealed the relative abundance of phosphorylated OCT‐4 peptides that contain putative PIM1 phosphorylation at S289 and S290, suggesting that PIM1 phosphorylates OCT‐4 (Brumbaugh *et al.*, 2012). PIM kinase inhibitor treatment could block this phosphorylation inhibiting the ability of OCT‐4 to work synergistically with SOX2. Consequently, the decreased binding of these two transcription factors could reduce H19 levels.

We showed that expression of PIM1 in T‐ALL modulates the methylation status of H19 DMR, suggesting that the PIM kinase could act directly or indirectly as an epigenetic regulator. It has been suggested that PIM regulates DNA methylation. Phosphorylation of heterochromatin protein 1γ (HP1) at Ser‐93 by PIM1 promotes HP1 binding with histone H3K9me3, which leads to heterochromatin formation and the suppression of gene transcription responsible for proliferation (Jin *et al.*, 2014). Further experiments are required to decipher the exact mechanism by which PIM controls H19 levels.

Prostate cancer cells can escape ADT through a change in lineage identity driven by elevated SOX2. Knocking down SOX2 can restore sensitivity to Enza *in vitro* and in mouse xenograft models (Mu *et al.*, 2017). Our findings are consistent with these observations in that small molecule PIM‐i cells downregulate SOX2 and sensitize androgen‐responsive LNCaP cells to Enza therapy. This result could be secondary to the ability of these inhibitors to decrease H19. A recent study (Lawrence *et al.*, 2018) showed that the combination of pan‐PIM‐i and RNA polymerase I inhibitor targeting ribosomal biosynthesis was effective against all four neuroendocrine‐like AR‐null patient‐derived xenografts. These tumor cells exhibited heterogeneous mechanisms of resistance, including AR mutations and genomic structural rearrangements of the AR gene (Lawrence *et al.*, 2018).

## 5 Conclusions

Our data demonstrate that PIM kinase induction increases the lncRNA H19 and this in turn regulates stem cell genes, including the transcription factor SOX2, which plays a role in controlling the response to ADT. Additionally, we found that while elevated levels of PIM1 contribute resistance to ADT, the combination of a pan‐PIM‐i and H19 KD can reduce the tumor‐forming capacity of highly aggressive NEPC. Together, these data suggest a novel pathway controlled by the PIM kinases whose inhibition could impact clinical outcomes.

## Conflict of interest

The authors declare no conflict of interest.

## Author contributions

NS, VO and ASK wrote the manuscript. NS, SKRP, JJB, RP, JHS, and VO designed and carried out the experiments. KO and HB supplied essential reagents. NS, VO and ASK conceived the project.

## Supporting information


**Fig. S1.** Increased expression of PIM2 in PIM‐i sensitive vs resistant T‐ALL.

**Fig. S2.** PIM1 decreased the DNA methylation of H19 DMR in SUPT1 cells.

**Fig. S3.** PIM1 overexpression induces a stem cell like phenotype in PC3 cells.

**Fig. S4.** c‐MYC expression has no effect on H19 levels.

**Fig. S5.** Pharmacological inhibition of PIM kinases reduces stem cell gene expression in NEPC cell line.

**Fig. S6.** PIM1 overexpression restores partial sensitivity in PIM‐i resistant T‐ALL cells.

**Fig. S7.** PIM kinase is highly expressed in NEPC.

**Fig. S8.** KD of H19 expression in NEPC organoid OWCM‐155.

**Fig. S9.** Synergistic effect of pan‐PIM‐i with Enza in LNCaP/H19.

**Table S1.** Microarray expression analysis of six T‐ALL cell lines.

**Table S2.** Pathway analysis of transcriptomes from Microarray data from six T‐ALL cell lines.
