# Supplementary material for: PIM protein kinases regulate the level of the long noncoding RNA H19 to control stem cell gene transcription and modulate tumor growth
Source: Mol Oncol. 2020 Apr 1;14(5):974–90. doi: 10.1002/1878-0261.12662 (PMC7191193; doi:10.1002/1878-0261.12662)
Supplement: Supplementary file 1 — Fig. S1. Increased expression of PIM2 in PIM‐i sensitive vs resistant T‐ALL. [file MOL2-14-974-s001.pdf]

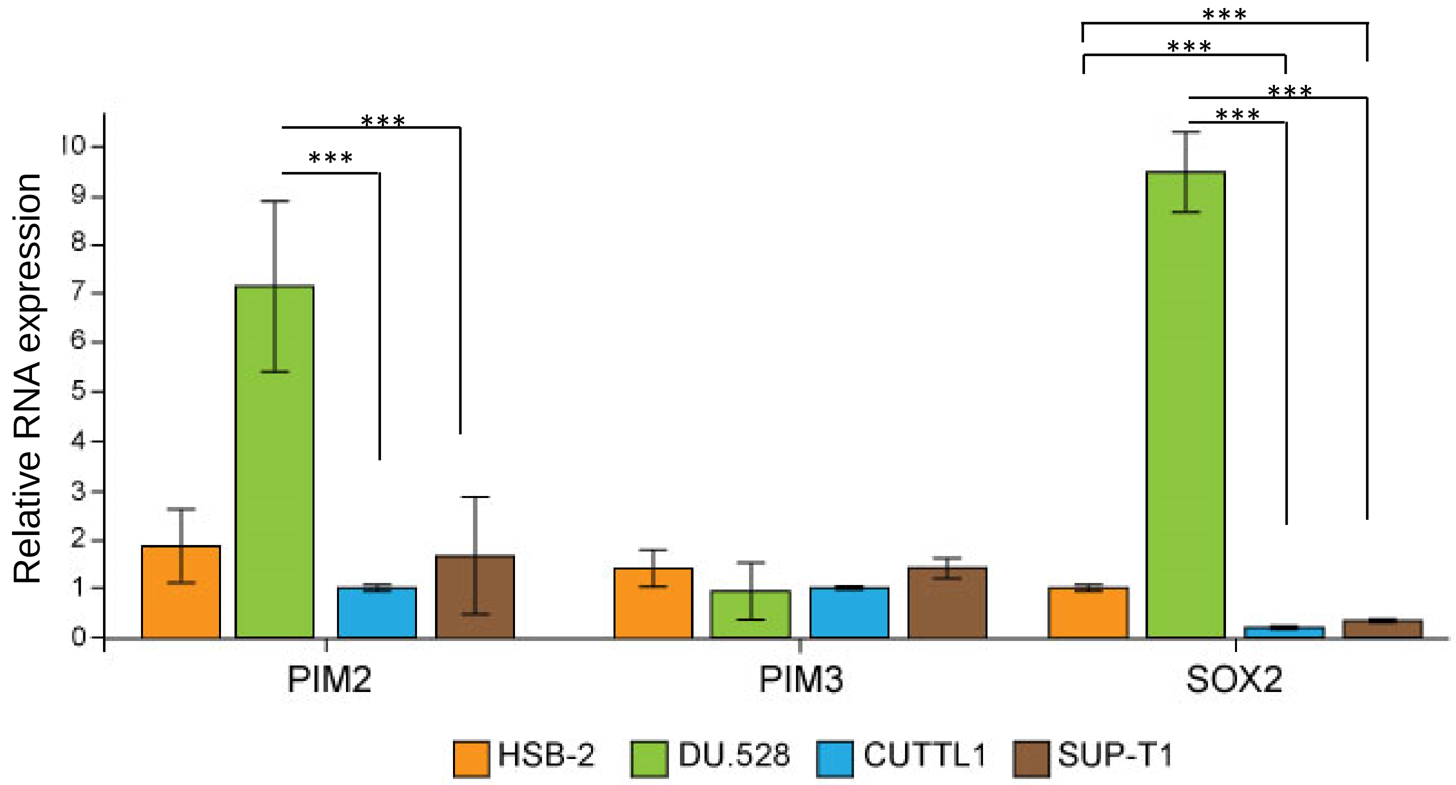

Figure S1: Increased expression of PIM2 in PIM-i sensitive vs resistant T-ALL. Relative mRNA expression of PIM2, PIM3 and SOX2 in indicated T-ALL cell lines. RNA expression are normalized to 18S RNA. Data are mean +/- S.D., n=3, ***p<0.001.
