# Supplementary material for: PIM protein kinases regulate the level of the long noncoding RNA H19 to control stem cell gene transcription and modulate tumor growth
Source: Mol Oncol. 2020 Apr 1;14(5):974–90. doi: 10.1002/1878-0261.12662 (PMC7191193; doi:10.1002/1878-0261.12662)
Supplement: Supplementary file 2 — Fig. S2. PIM1 decreased the DNA methylation of H19 DMR in SUPT1 cells. [file MOL2-14-974-s002.pdf]

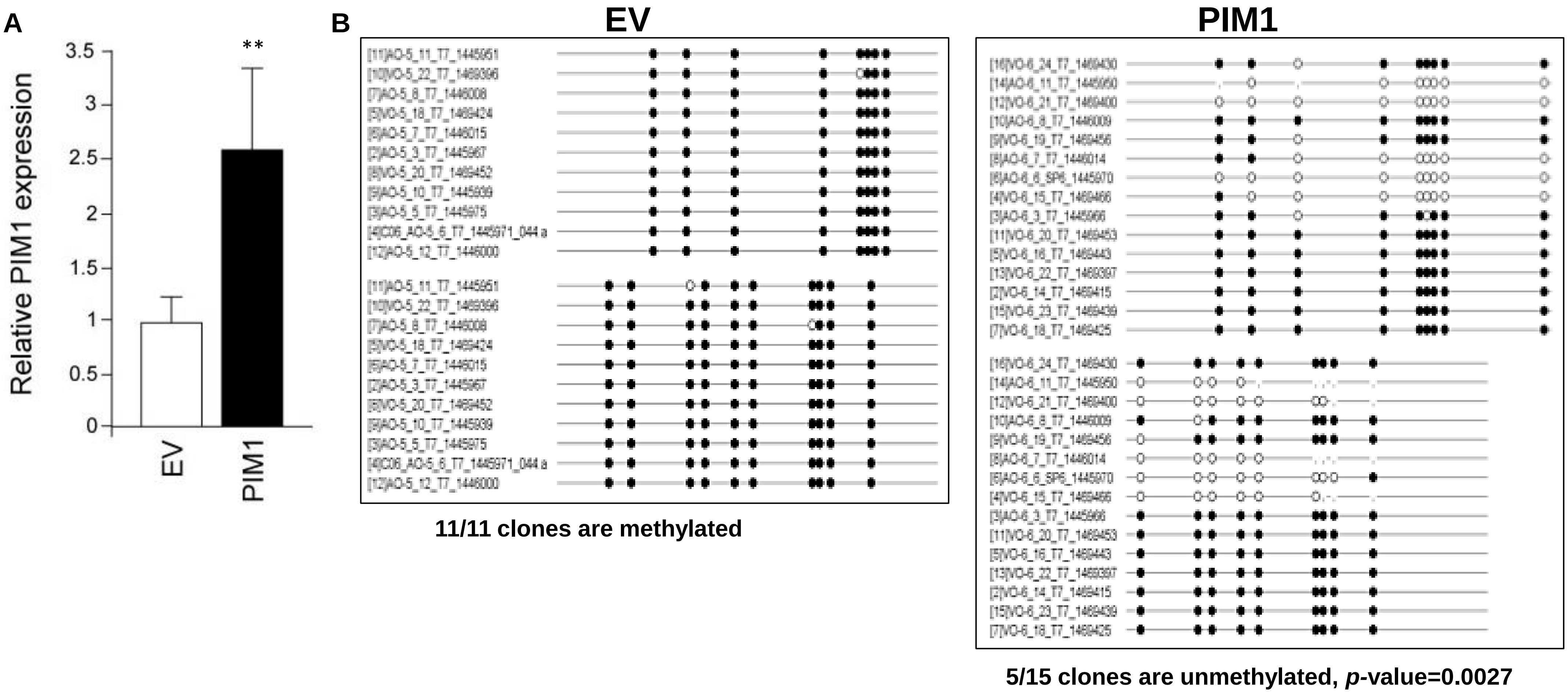

Figure S2: PIM1 decreased the DNA methylation of H19 DMR in SUPT1 cells. A. Relative H19 RNA expression after PIM1 overexpression in SUP-T1E cells versus control vector expressing cells. RNA levels were normalized to 18S RNA. Data are mean +/- S.D., n=3, **p<0.01. **B.** Bisulfite sequencing of H19 DMR in SUP-T1E cells overexpressing PIM1 or an empty vector (as control). Eleven SUP-T1E control clones and fifteen overexpressing PIM1 clones were sequenced. Dark circles represent methylated CpG and open circles unmethylated CpG.
