# Supplementary material for: PIM protein kinases regulate the level of the long noncoding RNA H19 to control stem cell gene transcription and modulate tumor growth
Source: Mol Oncol. 2020 Apr 1;14(5):974–90. doi: 10.1002/1878-0261.12662 (PMC7191193; doi:10.1002/1878-0261.12662)
Supplement: Supplementary file 3 — Fig. S3. PIM1 overexpression induces a stem cell like phenotype in PC3 cells. [file MOL2-14-974-s003.pdf]

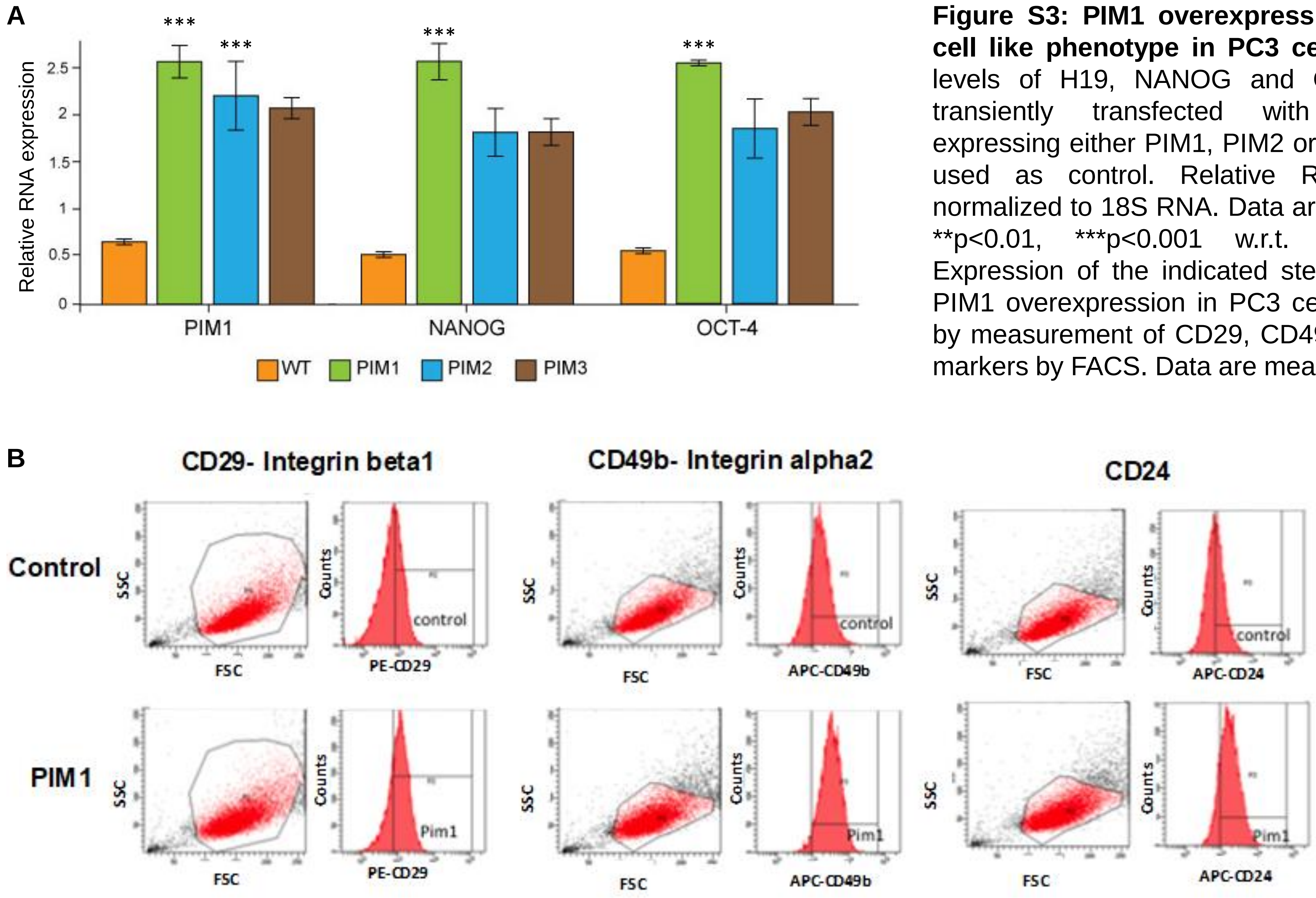

Figure S3: PIM1 overexpression induces a stem cell like phenotype in PC3 cells. A, Relative RNA levels of H19, NANOG and OCT-4 in PC3 cells transiently transfected with FUCRW vectors expressing either PIM1, PIM2 or PIM3. WT cells were used as control. Relative RNA expression are normalized to 18S RNA. Data are mean +/- S.D., n=3, **p<0.01, ***p<0.001 w.r.t. controls (WT). **B**, Expression of the indicated stem cell markers upon PIM1 overexpression in PC3 cells followed 48h later by measurement of CD29, CD49b and CD24 surface markers by FACS. Data are mean +/- S.D., n=3.
