# Supplementary material for: PIM protein kinases regulate the level of the long noncoding RNA H19 to control stem cell gene transcription and modulate tumor growth
Source: Mol Oncol. 2020 Apr 1;14(5):974–90. doi: 10.1002/1878-0261.12662 (PMC7191193; doi:10.1002/1878-0261.12662)
Supplement: Supplementary file 4 — Fig. S4. c‐MYC expression has no effect on H19 levels. [file MOL2-14-974-s004.pdf]

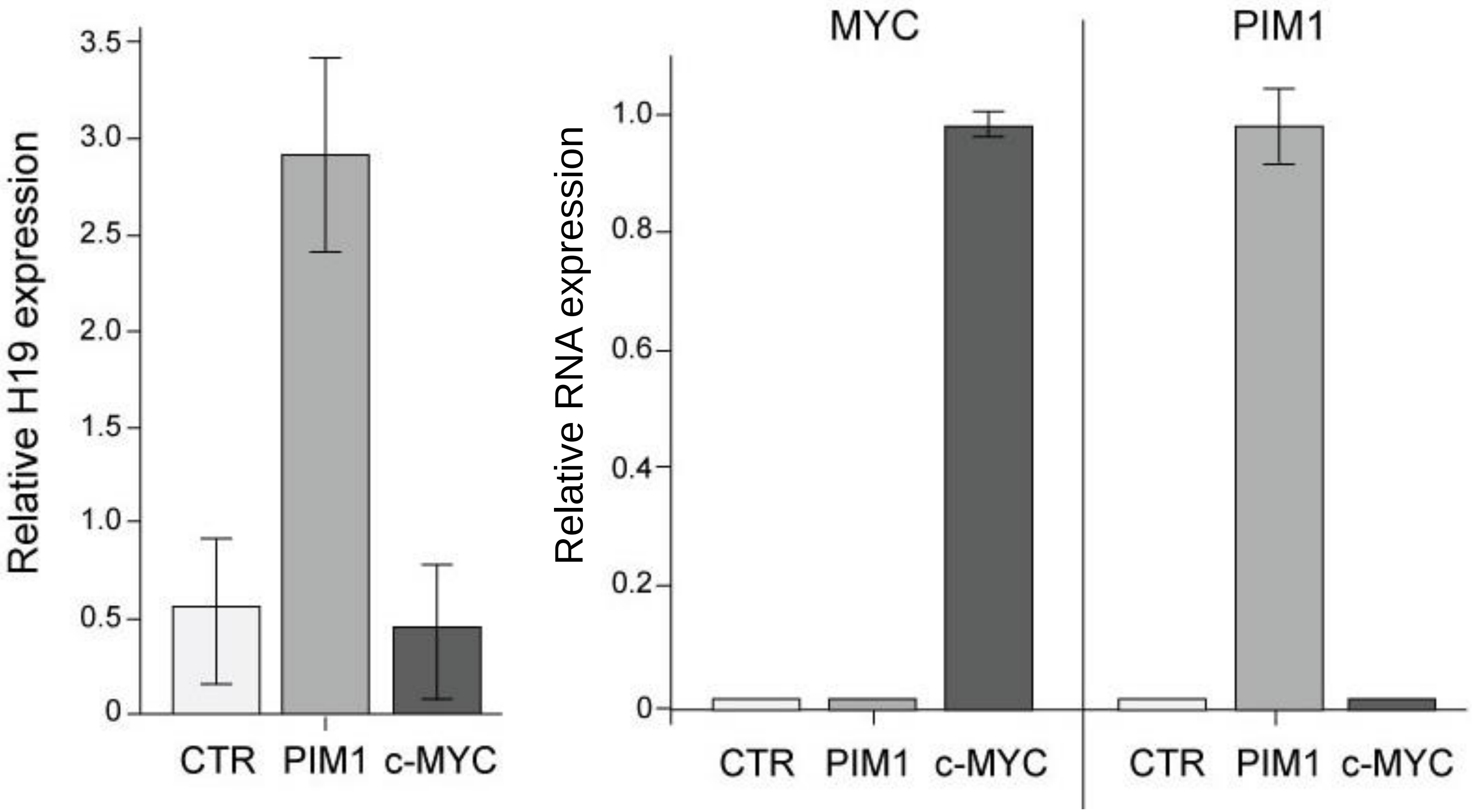

Figure S4: c-MYC expression has no effect on H19 levels. PIM1 or c-MYC were transiently overexpressed in DU145 cells. H19 levels are shown after PIM1 or c-MYC transfection. RNA level are normalized to 18S RNA (left panel). PIM1 and c-MYC RNA levels measured after transfection (right panel). Data are mean +/- S.D., n=3, **p<0.01, ***p<0.001 control (CTR).
