# Supplementary material for: PIM protein kinases regulate the level of the long noncoding RNA H19 to control stem cell gene transcription and modulate tumor growth
Source: Mol Oncol. 2020 Apr 1;14(5):974–90. doi: 10.1002/1878-0261.12662 (PMC7191193; doi:10.1002/1878-0261.12662)
Supplement: Supplementary file 5 — Fig. S5. Pharmacological inhibition of PIM kinases reduces stem cell gene expression in NEPC cell line. [file MOL2-14-974-s005.pdf]

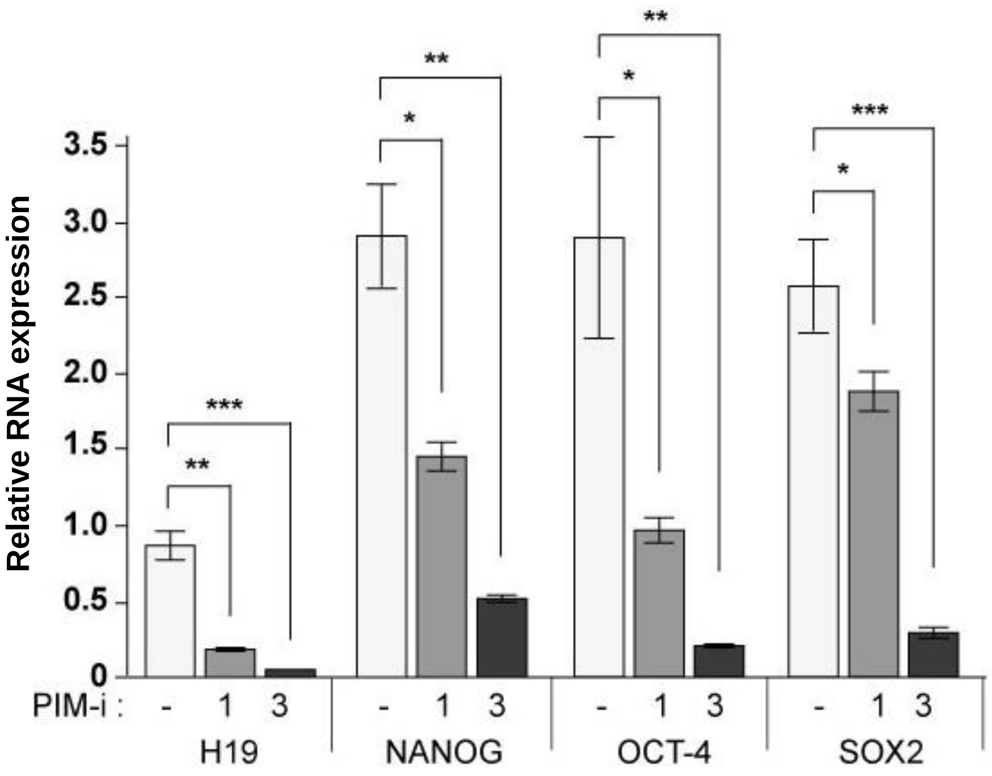

Figure S5: Pharmacological inhibition of PIM kinases reduces stem cell gene expression in NEPC cell line. Relative mRNA expression of H19, NANOG, OCT-4 and SOX2 in neuroendocrine prostate cancer (NEPC) cell line LASCPC-01 treated with DMSO or PIM-i (PIM447, 1µM and 3µM). RNA expression are normalized to 18S RNA. Data are mean +/- S.D., n=3, *p<0.05, **p<0.01, ***p<0.001.
