# Supplementary material for: PIM protein kinases regulate the level of the long noncoding RNA H19 to control stem cell gene transcription and modulate tumor growth
Source: Mol Oncol. 2020 Apr 1;14(5):974–90. doi: 10.1002/1878-0261.12662 (PMC7191193; doi:10.1002/1878-0261.12662)
Supplement: Supplementary file 6 — Fig. S6. PIM1 overexpression restores partial sensitivity in PIM‐i resistant T‐ALL cells. [file MOL2-14-974-s006.pdf]

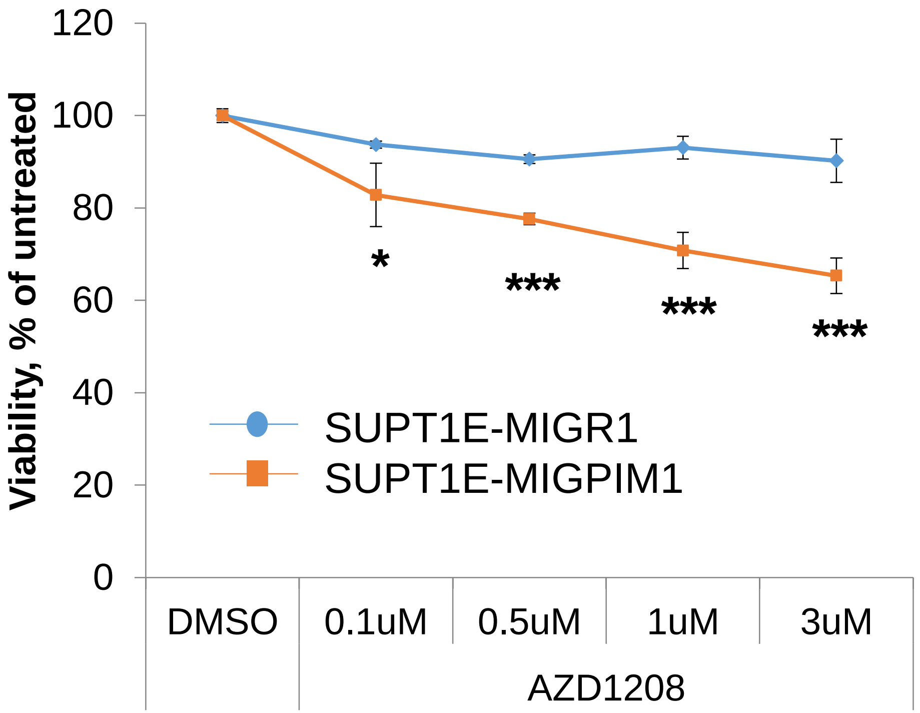

Figure S6: PIM1 overexpression restores partial sensitivity in PIM-i resistant T-ALL cells. Percent viability of SUPT1E cells after MIGR1 and MIGPIM1 transduction, treated with PIM-i (AZD1208) at the indicated doses. DMSO was used as control. Data are mean +/- S.D., n=3, *$p<0.05$, **$p<0.01$, ***$p<0.001$ vs. SUP-T1E-MIGR1.
