# Supplementary material for: PIM protein kinases regulate the level of the long noncoding RNA H19 to control stem cell gene transcription and modulate tumor growth
Source: Mol Oncol. 2020 Apr 1;14(5):974–90. doi: 10.1002/1878-0261.12662 (PMC7191193; doi:10.1002/1878-0261.12662)
Supplement: Supplementary file 7 — Fig. S7. PIM kinase is highly expressed in NEPC. [file MOL2-14-974-s007.pdf]

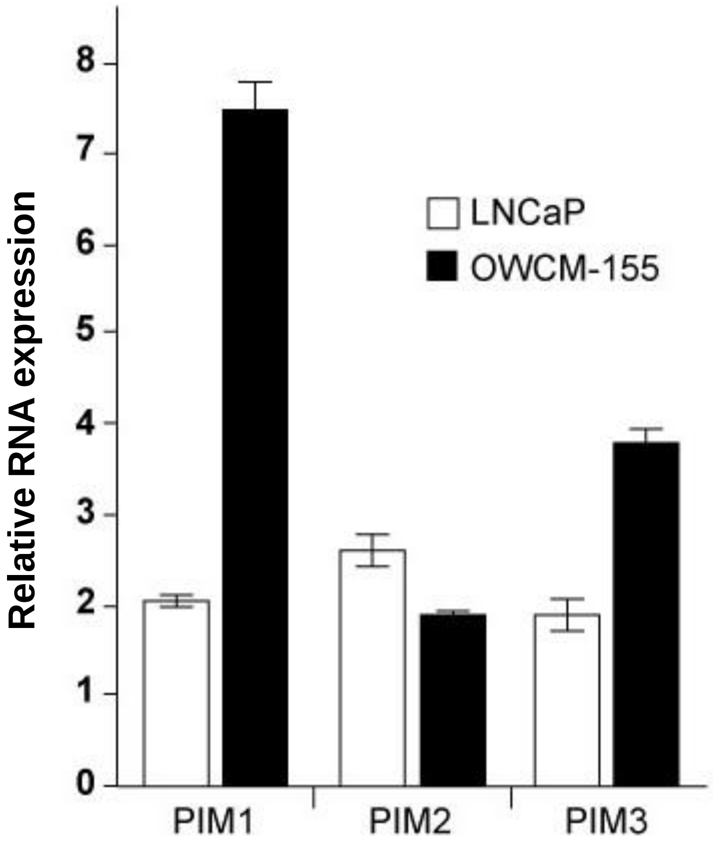

Figure S7: PIM kinase is highly expressed in NEPC. Relative RNA expression of PIM1/2/3 in OWCM-155 NEPC organoids and androgen responsive adenocarcinoma cell line LNCaP. RNA expression are normalized to 18S RNA. Data are mean +/- S.D., n=3.
