# Supplementary material for: PIM protein kinases regulate the level of the long noncoding RNA H19 to control stem cell gene transcription and modulate tumor growth
Source: Mol Oncol. 2020 Apr 1;14(5):974–90. doi: 10.1002/1878-0261.12662 (PMC7191193; doi:10.1002/1878-0261.12662)
Supplement: Supplementary file 8 — Fig. S8. KD of H19 expression in NEPC organoid OWCM‐155. [file MOL2-14-974-s008.pdf]

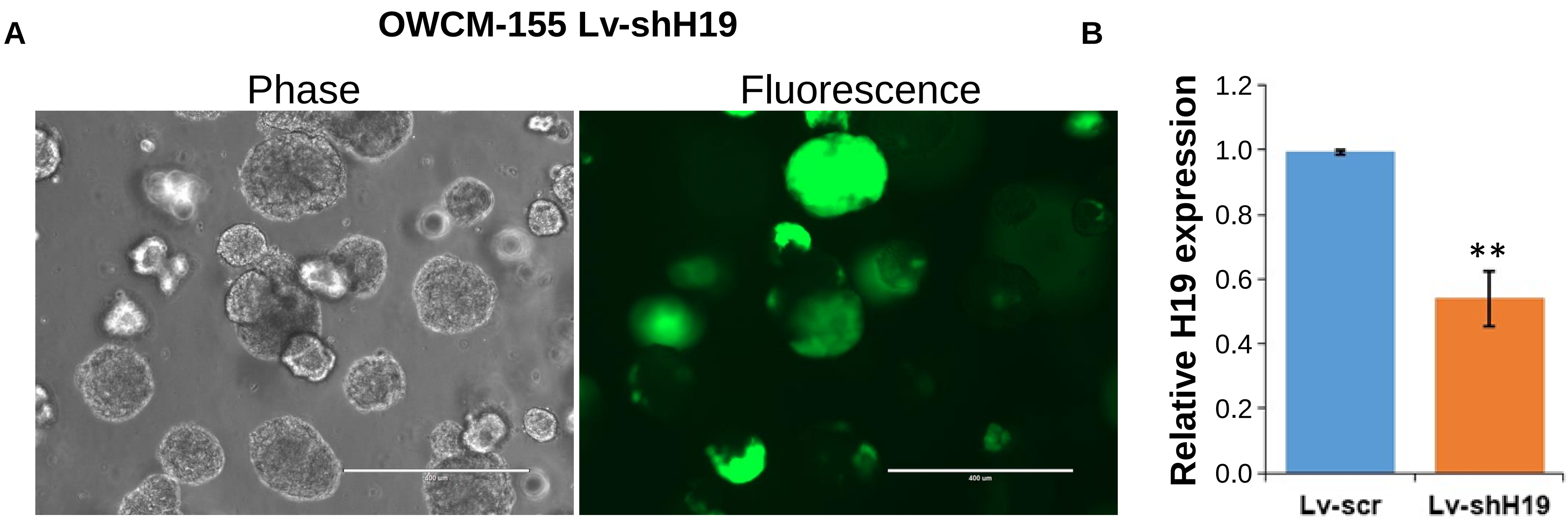

Figure S8: Knockdown of H19 expression in NEPC organoid OWCM-155. A, Representative images of lentiviral transduction of OWCM-155 with GFP expressing lentiviral plasmid pLenti-siH19-GFP, scale bar: 400µm. **B.** Relative H19 RNA expression in OWCM-155 transduced with control (Lv-scr) or H19 KD (Lv-shH19) lentivector. Relative H19 expression was normalized to 18S RNA. Values are mean +/- S.D. ** represents p-value <0.01.
