# Supplementary material for: PIM protein kinases regulate the level of the long noncoding RNA H19 to control stem cell gene transcription and modulate tumor growth
Source: Mol Oncol. 2020 Apr 1;14(5):974–90. doi: 10.1002/1878-0261.12662 (PMC7191193; doi:10.1002/1878-0261.12662)
Supplement: Supplementary file 9 — Fig. S9. Synergistic effect of pan‐PIM‐i with Enza in LNCaP/H19. [file MOL2-14-974-s009.pdf]

A

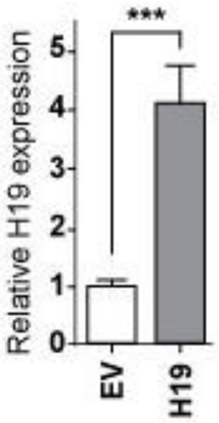

B

Dose ENZA	Dose PIM-i	Effect	Combination Index
2.0 μM	0.3 μM	0.52962	0.66509
2.0 μM	1.0 μM	0.63415	0.66455
5.0 μM	0.3 μM	0.80662	0.35645
5.0 μM	1.0 μM	0.84321	0.34847

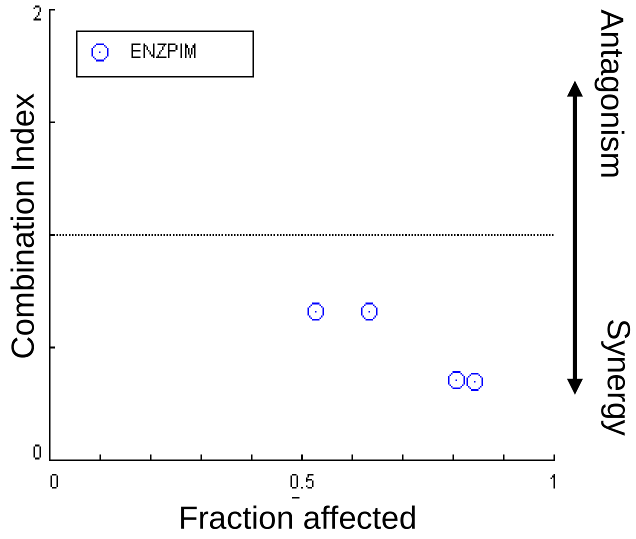

Figure S9: Synergistic effect of pan-PIM inhibitor(s) with Enzalutamide in LNCaP/H19. A, Relative H19 levels in LNCaP cells transduced with EV and H19. RNA expression is normalized to 18S RNA. Data are mean +/- S.D., n=3, ***p<0.001. **B**, LNCaP and LNCaP/H19 cells were treated with PIM447 either alone or in combination with Enza for 72 h and percentage viable cells was determined by XTT assay. The percent growth inhibition of drugs alone and in combination was determined and the combination index (CI) for Enza 2μM/ 5μM at the indicated concentrations of PIM447 was determined by Combosyn plot. The combination index value of below 1 indicates synergism.
